# Supplementary material for: Trajectories of CD4+/CD8+ T-Cells Ratio 96 Weeks after Switching to Dolutegravir-Based Two-Drug Regimens: Results from a Multicenter Prospective Cohort Study
Source: Viruses. 2022 Oct 22;14(11):2315. doi: 10.3390/v14112315 (PMC9696165; doi:10.3390/v14112315)
Supplement: Supplementary file 1 [file viruses-14-02315-s001.zip › viruses-1934844-supplementary.pdf]

Table S1: Details of previous and current antiretroviral therapy (ART) exposure of study participants.

	Total	DTG+3TC	DTG+PI	DTG+RPV	DTG+ TDF/FTC	DTG+ TAF/FTC	DTG+ ABC/3TC	P
	N=533	N=120 (22.5%)	N=38 (7.1%)	N=67 (12.6%)	N=49 (9.2%)	N=27 (5.1%)	N=232 (43.5%)	
**Time on ART (years), median (IQR)**	10.6 (4.8-18.1)	9.9 (4.9-15.5)	18.1 (7.2-20.2)	13.7 (7.0-21.8)	11.2 (3.3-18.1)	11.3 (5.7-17.7)	9.2 (4.4-16.5)	0.002
**Drug classes in previous ART**								
**NRTI**	507 (95.1)	118 (98.3)	36 (94.7)	63 (94.0)	221 (95.3)	43 (87.8)	26 (96.3)	0.89
median months (IQR)	96 (48-176)	96 (51-157)	148 (80-210)	132 (64-208)	84 (44-166)	78 (42-160)	132 (64-199)	0.03
**NNRTI**	318 (59.7)	64 (53.3)	24 (63.2)	54 (80.6)	136 (58.6)	23 (46.9)	17 (63.0)	0.002
median months (IQR)	54 (28-119)	52 (38-121)	44 (22-72)	74 (40-130)	53 (26-97)	56 (16-150)	105 (36-155)	0.28
**PI**	391 (73.4)	83 (69.2)	35 (92.1)	53 (79.1)	162 (69.8)	36 (73.5)	21 (77.8)	0.008
median months (IQR)	70 (35-119)	69 (36-96)	98 (61-164)	79 (39-137)	64 (29-120)	79 (36-105)	30 (20-104)	0.02
**INSTI**	14 (2.6)	5 (4.2)	0	8 (11.9)	0	0	0	<0.0001
median months (IQR)	39 (11-71)	29 (14-51)	-	60 (27-90)	-	-	-	0.31
**Other**	204 (38.3)	58 (48.3)	20 (52.6)	38 (56.7)	66 (28.4)	11 (22.4)	11 (40.7)	<0.0001
median months (IQR)	32 (13-54)	26 (11-50)	39 (16-61)	44 (23-80)	21 (8-44)	44 (12-60)	64 (17-69)	0.005
**Overall classes**								
2	209 (39.2)	48 (40.0)	8 (21.0)	12 (17.9)	108 (46.6)	23 (46.9)	10 (37.0)	
3	204 (38.3)	52 (43.3)	13 (34.2)	22 (32.8)	91 (39.2)	17 (34.7)	9 (33.3)	
4-5	99 (18.6)	19 (15.8)	15 (39.5)	30 (44.8)	24 (10.3)	4 (8.2)	7 (25.9)	
unclear	21 (3.9)	1 (0.8)	2 (5.3)	3 (4.5)	9 (3.9)	5 (10.2)	1 (3.7)	<0.0001
**Time on last regimen before enrolment (months), median (IQR)**	30 (16-56)	28 (18-41)	24 (12-51)	30 (15-47)	34 (13-77)	24 (17-56)	32 (15-69)	0.92
**PI on last regimen, n (%)**	248 (46.5)	53 (44.1)	30 (79.0)	20 (29.8)	25 (51.0)	12 (44.4)	108 (46.5)	0.0002
ATV	109 (41.1)	27 (22.5)	7 (10.4)	7 (10.4)	8 (16.3)	6 (22.2)	57 (24.5)	0.08
DRV	115 (46.4)	25 (20.8)	23 (60.5)	13 (19.4)	11 (22.4)	5 (18.5)	38 (16.3)	<0.0001
**INSTI on last regimen, n (%)**	103 (19.3)	23 (19.1)	14 (36.8)	18 (26.8)	5 (10.2)	6 (22.2)	37 (15.9)	0.01
RAL	72 (13.5)	12(2.2)	13 (34.2)	16 (23.88)	5 (10.2)	4 (14.8)	22 (9.4)	0.0001
EVG	31 (5.8)	11 (9.1)	1 (2.6)	2 (2.9)	0 (0.0)	2 (7.4)	15 (6.4)	0.19
**NNRTI on last regimen, n (%)**	184 (34.5)	28 (23.3)	9 (23.7)	43 (64.1)	17 (34.6)	11 (40.7)	76 (32.7)	<0.0001
EFV	65 (12.2)	11 (9.1)	2 (7.1)	3 (4.4)	10 (20.4)	3 (11.1)	36 (15.5)	0.045
RPV	62 (11.6)	10 (8.3)	4 (10.5)	23 (34.3)	3 (6.1)	5 (18.5)	17 (7.3)	<0.0001
NVP	41 (7.7)	5 (4.1)	2 (5.3)	11 (16.4)	3 (6.1)	2 (7.4)	18 (7.7)	0.08
**NRTI on last regimen, n (%)**	440 (82.6)	106 (88.3)	20 (52.6)	40 (59.7)	42 (85.7)	26 (96.3)	206 (88.7)	<0.0001
TAF	61 (11.4)	26 (21.7)	2 (5.3)	6 (9.0)	0 (0.0)	13 (48.2)	14 (6.0)	<0.0001
TDF	160 (30.0)	21 (17.5)	14 (36.8)	15 (22.3)	36 (73.4)	8 (29.6)	66 (28.4)	<0.0001
FTC	217 (40.7)	45 (62.5)	14 (42.1)	21 (31.3)	35 (71.4)	21 (77.7)	79 (34.0)	<0.0001
ABC	174 (32.6)	25 (20.8)	4 (10.5)	14 (20.9)	3 (6.1)	2 (7.4)	126 (54.3)	<0.0001
3TC	221 (41.4)	60 (50.0)	4 (10.5)	18 (26.8)	6 (12.2)	5 (18.5)	128 (55.1)	<0.0001
**Time on current regimen (months), median (IQR)**	27 (13-49)	20 (13-25)	48 (26-56)	21 (9-50)	51 (38-61)	11 (6-19)	39 (14-50)	<0.0001
